# Arthroscopic debridement and microfracture for bilateral osteochondral lesions on the lateral process of the talus involving the subtalar joint: A case report

**DOI:** 10.1097/MD.0000000000038302

**Published:** 2024-05-24

**Authors:** Hiroki Yabiku, Tomohiro Matsui, Takeshi Sugimoto, Hideaki Nagamoto, Yasunori Tome, Kotaro Nishida, Tsukasa Kumai

**Affiliations:** a Department of Orthopedic Surgery, Graduate School of Medicine, University of the Ryukyus, Okinawa, Japan; b Department of Orthopaedic Surgery, Saiseikai Nara Hospital, Nara, Japan; c Department of Orthopedic Surgery, Osaka Global Orthopedic Hospital, Osaka, Japan; d Department of Orthopaedic Surgery, Tohoku University, Sendai, Miyagi, Japan; e Graduate School of Sports Sciences, Waseda University, Tokorozawa, Saitama, Japan; f Faculty of Sports Sciences, Waseda University, Tokorozawa, Saitama, Japan.

**Keywords:** accessory anterolateral talar facet, arthroscopic microfracture, lateral process of the talus, osteochondral lesion of the talus, subtalar joint

## Abstract

**Rationale:**

Osteochondral lesions on the lateral process of the talus involving the subtalar joint are rare; the optimal surgical treatment remains to be clarified as there are few reports. Additionally, bilateral cases are extremely rare. Therefore, the clinical outcomes of the surgical treatment for bilateral osteochondral lesions on the lateral process of the talus involving the subtalar joint have not been fully elucidated.

**Patient concerns:**

A 16-year-old boy who played soccer presented to our hospital with bilateral hindfoot pain. The symptoms persisted even after 3 months of conservative treatment. The patient and family requested surgical treatment to relieve the symptoms.

**Diagnoses:**

The patient was diagnosed with bilateral osteochondral lesions on the lateral process of the talus, involving the subtalar joint based on computed tomography and magnetic resonance imaging findings.

**Interventions:**

Arthroscopic debridement and microfracture were performed bilaterally.

**Outcomes:**

Postoperative computed tomography and magnetic resonance imaging of both feet revealed remodeling of the subchondral bone. The patient returned to play at the pre-injury level with no pain.

**Lessons:**

This report describes a case of bilateral osteochondral lesions on the lateral process of the talus, involving the subtalar joint. Arthroscopic debridement and microfracture were effective in relieving symptoms and the subchondral bone remodeling. To the best of our knowledge, this is the first report of arthroscopic treatment of osteochondral lesions of the lateral process of the talus involving the subtalar joint.

## 1. Introduction

Osteochondral lesions of the talus (OLTs), including osteochondral fracture of the talus and osteochondral dissecans, were first described by Canale and Belding.^[1]^ Etiologies of OLTs is thought to be multiple and controversial.^[2]^ Those are such as trauma including ankle sprains and fractures,^[3–5]^ avascular necrosis,^[6]^ degenerative change,^[7]^ or malalignment.^[8]^ Most of the affected lesions were located at the medial talar dome (62.8%) and subsequently at the lateral talar dome (33.4%).^[9]^ In either a pediatric/young or adult population with symptomatic OLTs, conservative therapy such as cast immobilization with non-weight-bearing or restriction of sports activities is considered a first-line treatment.^[10–13]^ In addition, hyaluronic acid or plate-rich plasma intra-articular injection for OLTs has been reported as a conservative treatment option.^[14–16]^ Surgical interventions, such as arthroscopic debridement/microfracture,^[17,18]^ retrograde drilling,^[19]^ fixation of osteochondral lesions,^[20,21]^ or osteochondral transplantation^[22,23]^ can be considered if immobilization/restriction of sports activities does not improve symptoms.

The OLT usually affects the posteromedial or anterolateral part of the talar dome,^[9]^ although it rarely occurs on the lateral process of the talus involving the subtalar joint.^[24–27]^ Previous reports described conservative treatment,^[24]^ open resection,^[25,26]^ or internal fixation.^[27]^ However, there is no consensus on treatment for OLT involving the subtalar joint and clinical outcomes of arthroscopic debridement and microfracture since it is a very rare disease with a few case reports.

In this case, we performed arthroscopic debridement and microfracture for bilateral OLTs involving the subtalar joint less invasively, and the patient achieved favorable short-term clinical outcomes.

## 2. Case report

**Figure 1.**
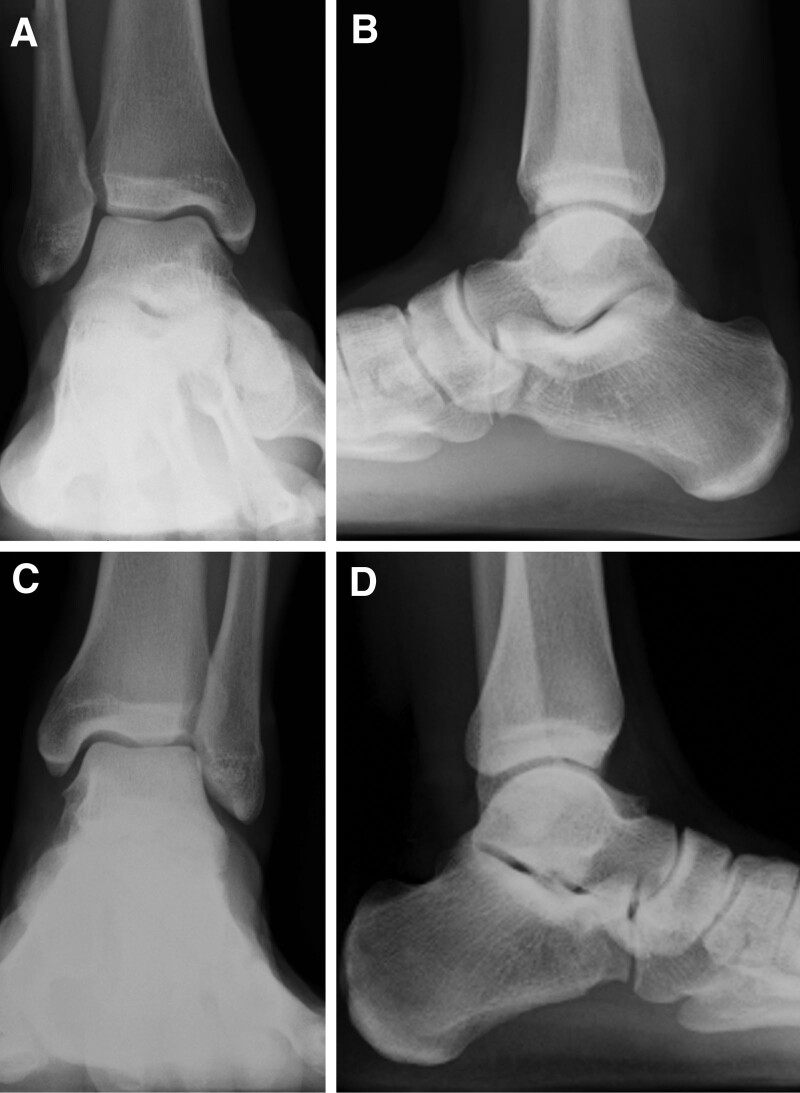
Preoperative radiographs of the right (A, B) and the left (C, D) feet show no obvious abnormal findings.

**Figure 2.**
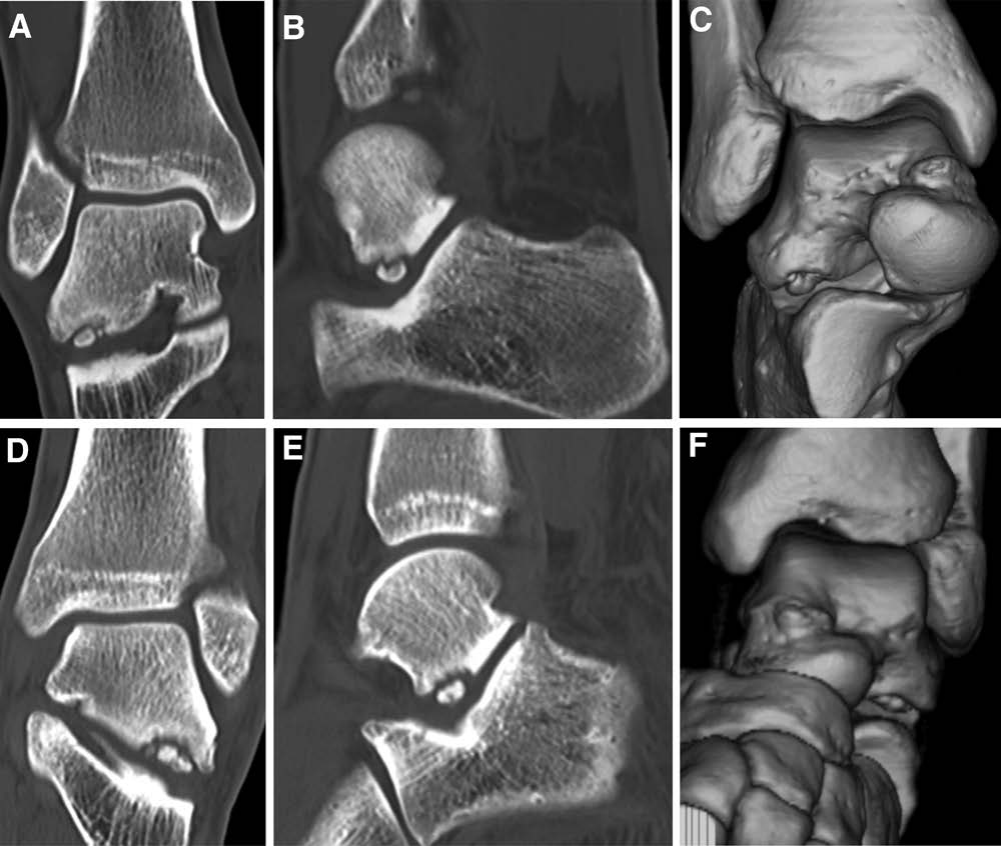
Preoperative computed tomography of the right (A–C) and the left (D–F) feet reveal bilateral osteochondral lesions on the lateral process of the talus involving the subtalar joint.

**Figure 3.**
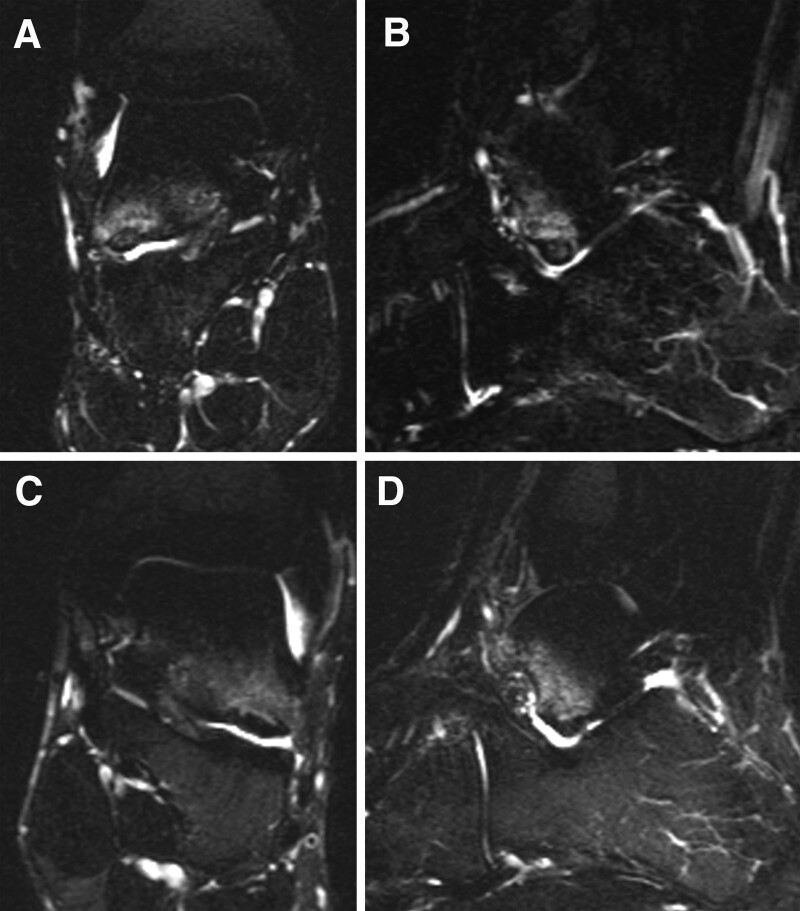
Preoperative magnetic resonance imaging of the right (A, B) and the left (C, D) feet reveal bone marrow edemas in the lateral process of the talus involving the subtalar joint; the lesion size was 6.9 × 6.8 mm in the right foot and 8.5 × 9.2 mm in the left foot (arrows).

**Figure 4.**
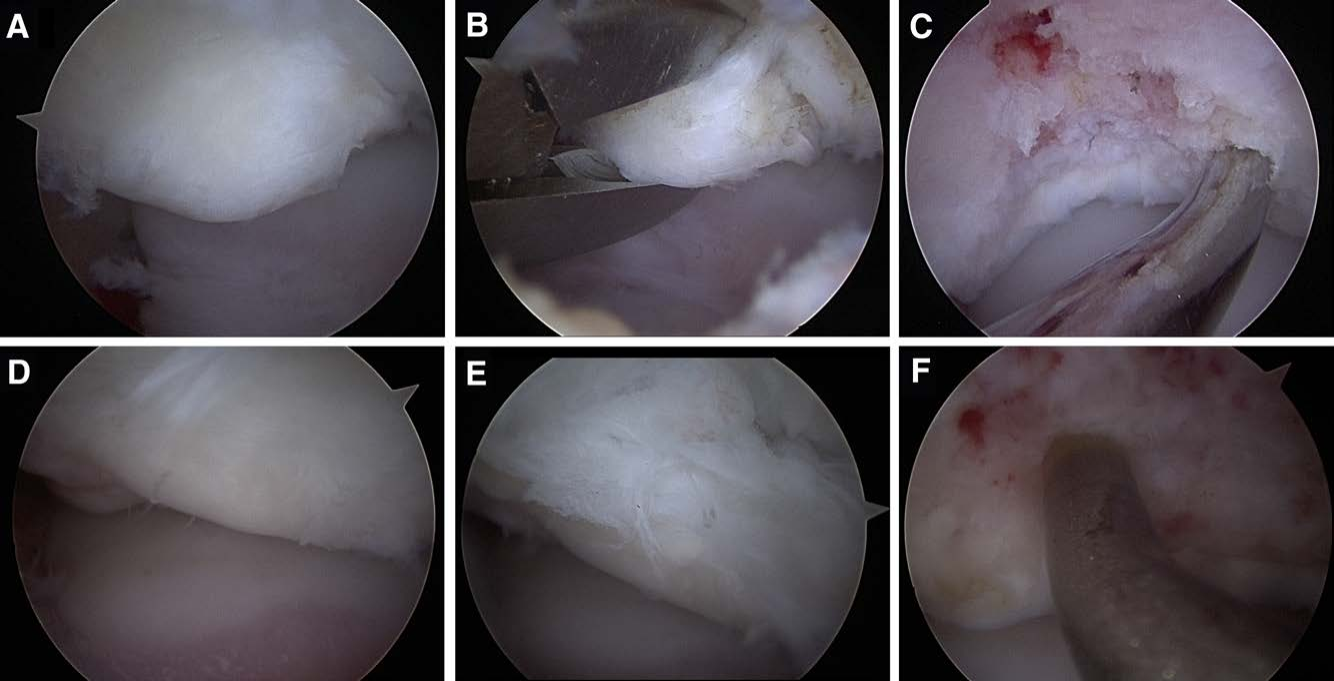
Intraoperative arthroscopic findings of the right (A–C) and the left (D–F) feet. Cartilaginous continuity between osteochondral lesion and surrounding articular surface was maintained (A, D). Probing reveal softening and instability of osteochondral lesions (B, E). Microfracture with awl, inducing subchondral bone bleeding (C, F).

**Figure 5.**
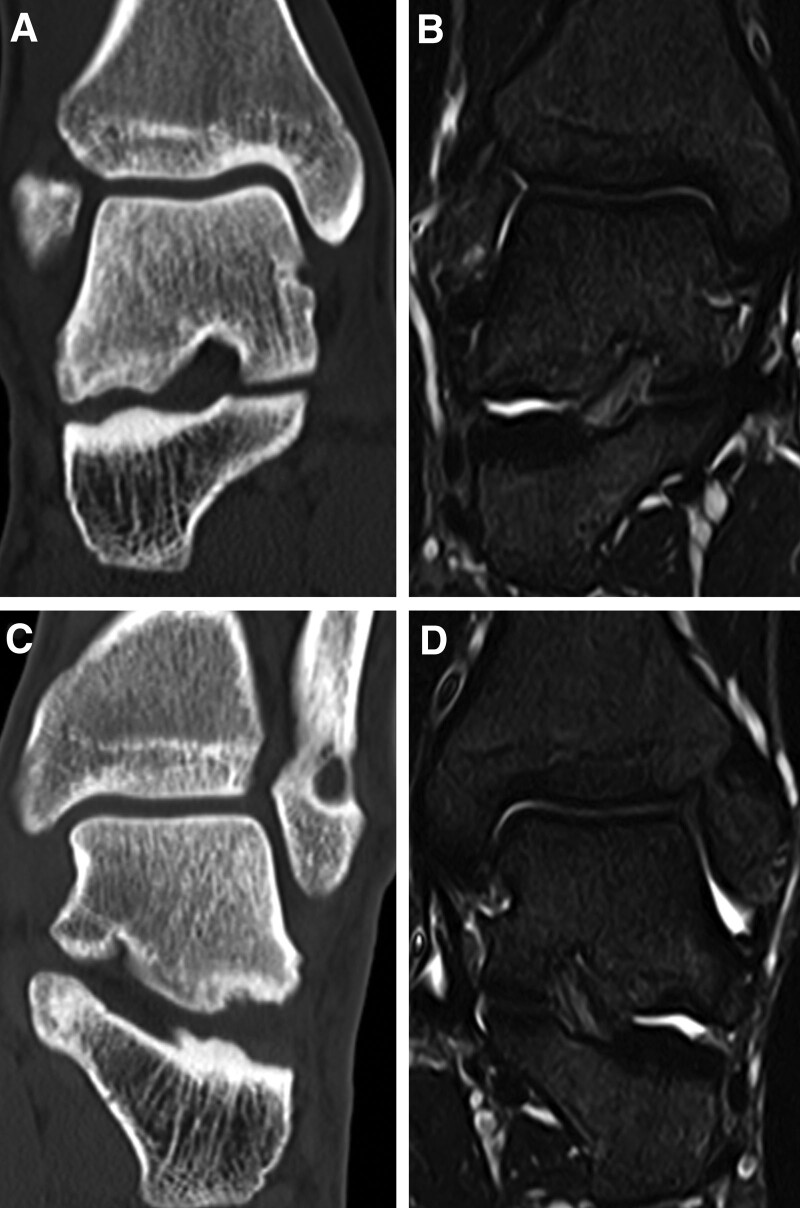
Postoperative computed tomography of the right (A) and the left (C) feet reveal remodeling of subchondral bones; magnetic resonance imaging of the right (B) and the left (D) feet reveal disappearances of bone mallow edemas on lateral process of the talus (arrows).

A 16-year-old male soccer player presented to our hospital with bilateral hindfoot pain. The patient twisted the right hindfoot while playing soccer. Conservative therapy, including a 3-week immobilization with non-weight-bearing and 3-month restriction of sports activities, was performed; however, the pain persisted. In addition, the patient experienced pain in the contralateral left hindfoot without any obvious causal events. The patient had no medical history, except for frequent bilateral ankle sprains. Physical examination revealed tenderness in the lateral process of the talus in both the feet. The range of motion of the bilateral hindfeet was normal, without instability. Plain radiographs showed no obvious abnormal findings (Fig. 1A–D). Computed tomography (CT) revealed bilateral osteochondral lesions on the lateral process of the talus involving the subtalar joint (Fig. 2A–F). Magnetic resonance imaging (MRI) revealed bilateral bone marrow edemas in the lateral process of the talus involving the subtalar joint; the lesion size was 6.9 × 6.8 mm in the right foot and 8.5 × 9.2 mm in the left foot (Fig. 3A–D). We decided to perform arthroscopic debridement and microfracture since the 3-month conservative treatment failed. The preoperative American Orthopaedic Foot and Ankle Society (AOFAS) ankle-hindfoot scale score^[28]^ was 87 points in both feet. Surgery of the right foot was performed, followed by 2 months later surgery of the left one. The patient was placed in the supine position under regional anesthesia and sciatic and saphenous nerve blocks were performed without a tourniquet. Traction was applied to increase the subtalar joint space. Arthroscopy was performed using 2 portals. A viewing portal was created proximal to the anterior process of the calcaneus. Another working portal was created just above the lateral edge of the fossa calcanei. Arthroscopic findings revealed an osteochondral lesion at the anterior edge of the posterior calcaneal articular surface of the talus. Moreover, cartilaginous continuity between the osteochondral lesion and the surrounding articular surface was maintained, although there was softening of the cartilage and instability that enabled us to determine the lesion. We removed the osteochondral lesion and performed a microfracture procedure with an awl until subchondral bone bleeding was confirmed (Fig. 4A–F). Postoperatively, partial weight-bearing and full weight-bearing were allowed at 3 and 6 weeks after surgery, respectively. Three months after left foot surgery, the patient returned to sports activity at the pre-injury level. Postoperative CT and MRI of both feet revealed remodeling of the subchondral bone and disappearance of bone mallow edema on the lateral process of the talus at the last follow-up 26 months after the first surgery (Fig. 5A–D). The patient returned to play soccer at the pre-injury level with no pain. The postoperative AOFAS ankle-hindfoot scale score was 100 points in both feet.

## 3. Discussion

In this case, we performed arthroscopic debridement and microfracture of bilateral osteochondral lesions of the talus involving the subtalar joint. To the best of our knowledge, this is the first report describing arthroscopic microfractures of bilateral osteochondral lesions of the talus involving the subtalar joint.

Although the etiology of OLT remains controversial,^[2]^ trauma or repetitive loading has been proposed as the cause.^[29,30]^ In fact, the present case had histories of frequent bilateral ankle sprains. OLT involving the subtalar joint has been reported in 5 feet in four cases^[24–27]^; all lesions in these cases were located at the anterior edge of the posterior calcaneal articular surface, which is similar to the area of fracture of the lateral process of the talus.^[31]^ Therefore, we thought that the mechanism of fracture of the lateral process of the talus, such as forced dorsiflexion with inversion and external rotation^[32]^ or eversion combined with dorsiflexion^[33]^ may cause OLT involving the subtalar joint. In this case, these types of minor sprains that do not lead to fractures of the lateral process of the talus may have caused the OLT. In addition, this case showed bilateral accessory anterolateral talar facet-like morphology. It has been suggested that the accessory anterolateral talar facet is associated with talocalcaneal impingement in an everted position.^[34]^ Kicking motion, which is often accomplished by striking the ball with the medial aspect of the foot and forefoot, is thought to place the foot everted and externally rotated.^[35]^ Moreover, Giza et al^[36]^ reported that, in soccer, the most common foot and ankle positions at the time of injury were pronated in the sagittal plane for weight-bearing limbs, and the most common foot and ankle rotations at the time of injury were external rotation and eversion. Based on these findings, the morphological features of this case and sports-specific motion of the foot and ankle may be the cause of the OLT.

Arthroscopic treatment, including debridement and microfracture, has been reported for OLTs.^[17,18,37]^ Jurina et al^[18]^ reported the clinical outcomes of arthroscopic microfractures for OLTs in skeletally immature patients. In this study, 76.9% of patients were evaluated as having good clinical results according to the Berndt and Harty outcome question.^[18]^ Regarding the size of OLTs, a recent review recommended that an OLTs of less than approximately 100 mm^2^ would be effective for bone marrow stimulation such as debridement and microfracture.^[38,39]^ In this case, the lesion size calculated by ellipse formula using MRI measured coronal and sagittal length are 37.1 mm^2^ in right and 61.8 mm^2^ in left, respectively, which are ideal for debridement and bone marrow stimulation. The lateral process of the talus is a challenging location in arthroscopic procedures. However, we could access the lateral process of the talus arthroscopically, which may be less invasive than open surgery. Retrograde drilling may also be effective for OLTs in this case; however, we did not select this because of the possibility of postoperative accessory anterolateral talar facet impingement.

In this case, favorable clinical outcomes could be achieved by arthroscopic debridement and microfracture, and the patient could return to sports activities even after short-term follow-up. Although there was no recurrence of OLTs in the lateral process of the talus involving the subtalar joint after 26 months of follow-up, the patient should continue with his follow-up sessions as there is a possibility of recurrence, secondary osteoarthritis, or other complications.

## 4. Conclusions

We describe the first case of bilateral OLTs on the lateral process of the talus involving the subtalar joint that was treated with arthroscopic microfracture. Arthroscopic debridement and microfracture effectively improved the patient’s symptoms on such these rare conditions.

## Acknowledgments

We would like to thank Dr Kotaro Higa, Dr Fuminari Uehara, Dr Takashi Toma, and Dr Chinatsu Azuma for their helpful discussions. We would also like to thank Editage (www.editage.com) and Paperpal Preflight (https://preflight.paperpal.com/jp/partner/wolterskluwer/medicine) for the English language editing.

## Author contributions

**Conceptualization:** Hiroki Yabiku, Yasunori Tome, Tsukasa Kumai.

**Data curation:** Hiroki Yabiku, Hideaki Nagamoto.

**Investigation:** Hiroki Yabiku, Yasunori Tome.

**Methodology:** Hiroki Yabiku, Tomohiro Matsui, Takeshi Sugimoto, Hideaki Nagamoto, Tsukasa Kumai.

**Project administration:** Hiroki Yabiku, Yasunori Tome, Tsukasa Kumai.

**Resources:** Hiroki Yabiku, Tomohiro Matsui, Takeshi Sugimoto, Hideaki Nagamoto, Tsukasa Kumai.

**Visualization:** Hiroki Yabiku.

**Writing – original draft:** Hiroki Yabiku, Yasunori Tome, Kotaro Nishida, Tsukasa Kumai.

**Supervision:** Yasunori Tome, Kotaro Nishida, Tsukasa Kumai.

**Writing – review & editing:** Yasunori Tome, Kotaro Nishida, Tsukasa Kumai.
